# Mid-Infrared Emissivity Retrieval from Nighttime Sentinel-3 SLSTR Images Combining Split-Window Algorithms and the Radiance Transfer Method

**DOI:** 10.3390/ijerph20010037

**Published:** 2022-12-20

**Authors:** Xin Ye, Huazhong Ren, Pengxin Wang, Zhongqiu Sun, Jian Zhu

**Affiliations:** 1 College of Information and Electrical Engineering, China Agricultural University, Beijing 100083, China; 2 Institute of Remote Sensing and Geographic Information System, School of Earth and Space Sciences, Peking University, Beijing 100871, China; 3 Academy of Forestry Inventory and Planning, National Forestry and Grassland Administration, Beijing 100714, China

**Keywords:** middle infrared, land surface emissivity, thermal infrared, remote sensing, split-window algorithm, Sentinel-3 SLSTR

## Abstract

Land surface emissivity is a key parameter that affects energy exchange and represents the spectral characteristics of land cover. Large-scale mid-infrared (MIR) emissivity can be efficiently obtained using remote sensing technology, but current methods mainly rely on prior knowledge and multi-temporal or multi-angle remote sensing images, and additional errors may be introduced due to the uncertainty of external data such as atmospheric profiles and the inconsistency of multiple source data in spatial resolution, observation time, and other information. In this paper, a new practical method was proposed which can retrieve MIR emissivity with only a single image input by combining the radiance properties of TIR and MIR channels and the spatial information of remote sensing images based on the Sentinel-3 Sea and land surface temperature radiometer (SLSTR) data. Two split-window (SW) algorithms that use TIR channels only and MIR and TIR channels to retrieve land surface temperature (LST) were developed separately, and the initial values of MIR emissivity were obtained from the known LST and TIR emissivity. Under the assumption that the atmospheric conditions in the local area are constant, the radiance transfer equations for adjacent pixels are iterated to optimize the initial values to obtain stable estimation results. The experimental results based on the simulation dataset and real SLSTR images showed that the proposed method can achieve accurate MIR emissivity results. In future work, factors such as angular effects, solar radiance, and the influence of atmospheric water vapor will be further considered to improve performance.

## 1. Introduction

Land surface emissivity is the ratio of the thermal radiance of the target to that of the blackbody with the same temperature, which represents the effectiveness of converting heat energy into radiant energy [1]. The mid-infrared (MIR) channel (3–5 μm) plays an important role in multiple application fields, including land surface classification [2], quantitative vegetation monitoring [3,4], and forest fire detection [5], and has the features of reduced atmospheric influence and greater variability of emissivity compared with the thermal infrared (TIR) spectrum [6,7], which has great potential to improve the performance of land surface temperature retrieval [8,9] and target detection [10].

MIR emissivity can be obtained efficiently based on remote sensing technology using multiple methods [11], which can be grouped into three categories: the kernel-driven bidirectional reflectance distribution function (BRDF) method [11,12,13], the temperature-independent spectral indices (TISI) method [14,15], and the temperature emissivity separation (TES) method [16,17]. The BRDF method calculates the MIR emissivity by the directional reflectance, which can be characterized using the semi-empirical BRDF to describe the non-Lambertian reflection behavior, based on Kirchhoff’s law [18]. The BRDF is constructed by combining different kernel functions for modeling, such as the Ross–Li model, assuming that the shapes of the function in the MIR spectral region are the same as those in the visible and near-infrared regions [7]. The fitting of BDRF parameters needs observation data with more than three angles of the same pixel, and only some remote sensing data sources can meet the requirements. The TISI method analyzes the emissivity spectra by combining the MIR and TIR channels under the assumption that the daytime TISI without solar radiance contribution is equal to the nighttime TISI. The bidirectional reflectance is estimated from the nighttime observation data to calculate the hemisphere-directional reflectance using an empirical model or BRDF model, and then the directional emissivity in the MIR channel is calculated based on Kirchhoff’s law [19,20]. The requirement for diurnal remote sensing image input not only limits the applicability of the algorithm but changes in the land surface or weather during the observation interval will also introduce more errors. The TES method can retrieve the emissivities of both TIR and MIR channels after atmospheric correction based on the relationship between the minimum emissivity and the maximum–minimum apparent emissivity difference (MMD) [21]. This method can be achieved with only a single remote sensing image, but the accuracy of the results strongly depends on the effect of atmospheric correction, which requires the high accuracy of the input external atmospheric parameters, such as synchronously measured or reanalysis atmospheric profiles, which is often difficult to guarantee.

It can be seen that although existing methods have achieved MIR emissivity estimation based on a variety of remote sensing data and successfully carried out several applications, there are still some limitations in practical use. For the TISI method, the variation of cloud coverage, precipitation, and other factors make the actual situation unable to meet the algorithm assumptions [22]; for the BRDF method, the different geometric information such as observation angles may make the image geometric registration more sensitive to noise [11]; and for the TES algorithm, although the MIR emissivity can be calculated from single-view images, the algorithm requires accurate atmospheric correction, and the observation of simultaneous and spatially continuous atmospheric parameters is hard to obtain [22]. Therefore, this paper aims to develop a new practical MIR emissivity estimation method that does not require additional data other than single-view remote sensing images.

The advantages of the split-window (SW) algorithm, which does not require atmospheric correction [23], and the stability of TIR emissivity [24] are fully exploited in the new method. SW algorithms using only TIR channels and both MIR and TIR channels were developed separately, and equations were established to obtain initial values of MIR emissivity using the principle that LST is independent of channels to calculate the initial values of MIR emissivity. Subsequently, assuming that the atmosphere is stable in the local area, the initial values were optimized using spatial information, which is the similarity of atmospheric conditions between neighboring pixels of the image due to their geographical proximity. The proposed method is applied to the simulation dataset and real images of Sentinel-3 SLSTR data with two TIR channels and one MIR channel observed at nighttime to avoid the influence of solar radiance on the MIR channel, respectively, and the accuracy verification results showed the effectiveness of the new method.

## 2. Data and Method

**Figure 1:**
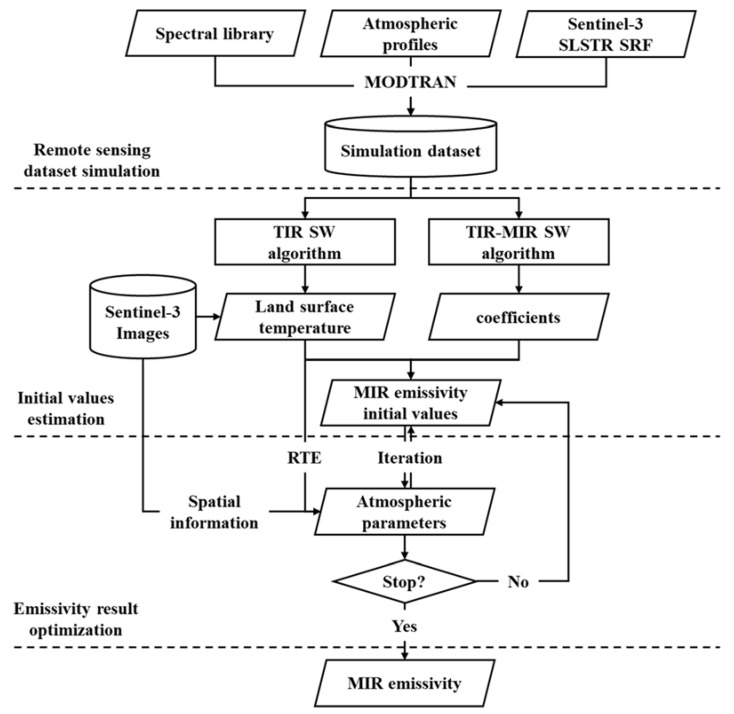
Flowchart of the proposed MIR emissivity retrieval method.

Figure 1 shows the flowchart of the proposed method, which includes three steps: (1) remote sensing dataset simulation, (2) initial values estimation, and (3) emissivity results optimization. First, the simulation dataset of the Sentinel SLSTR data is built using the MODTRAN model driven by the global atmospheric profiles and land spectral library. Then, the initial values of MIR emissivity are first obtained by the joint of the TIR SW algorithm and the MIR-TIR split-window algorithm. Finally, the retrieval results are obtained by iterative optimization with the spatial information provided by the neighboring pixels by assuming that the atmospheric parameters are constant in the local area of the Sentinel-3 image.

### 2.1. Remote Sensing Dataset Simulation

To develop the retrieval algorithm, a simulation dataset characterizing typical global land surface and atmospheric conditions is required. Therefore, 71 land surface samples, including 36 soil, 4 vegetation, 23 rock, 4 man-made, and 4 water samples, from ASTER [25] and UCSB [26] spectral libraries were selected to calculate the emissivity (*ε*) of Sentinel-3 MIR and TIR channels. Moreover, the atmospheric downward radiance, upward radiance, and transmittance (*L*_↓_, *L*_↑_, *τ*) were simulated by the moderate-spectral-resolution atmospheric transmittance (MODTRAN) model using the TIGR atmospheric profiles [27], which presented global typical atmospheric conditions, and the column water vapor (CWV) varies between 0.06 g/cm^2^ and 6.29 g/cm^2^. Considering the accuracy degradation of the SW algorithm under the humid atmosphere, this paper will base the analysis on relatively dry atmosphere conditions (CWV < 2.50 g/cm^2^) [22], with a total of 812 clear-sky global atmospheric profiles (global average CWV = 0.59 g/cm^2^), covering polar (regional average CWV = 0.23 g/cm^2^), mid-latitude (regional average CWV = 0.79 g/cm^2^), and tropical (regional average CWV = 1.69 g/cm^2^).

After obtaining the emissivity and atmospheric parameters, the observed radiance at the top of the atmosphere (*L_TOA_*) of the TIR and nighttime MIR channel can be calculated by the radiance transfer equation (RTE) shown in Equation (1):

(1) $$L_{TOA}=\left[\varepsilon \cdot B\left(T_{s}\right)+\left(1-\varepsilon \right)\cdot L_{↓}\right]\cdot \tau +L_{↑}$$

where *B*(.) is the Planck function and *T_s_* is the LST which is equal to the bottom air temperatures of the TIGR atmospheric profiles plus a temperature offset ([−10, 20] K with an interval of 5 K). In total, the simulation dataset in this paper contains 403,564 records (71 emissivities, 812 atmospheric profiles, and 7 LSTs).

**Figure 2:**
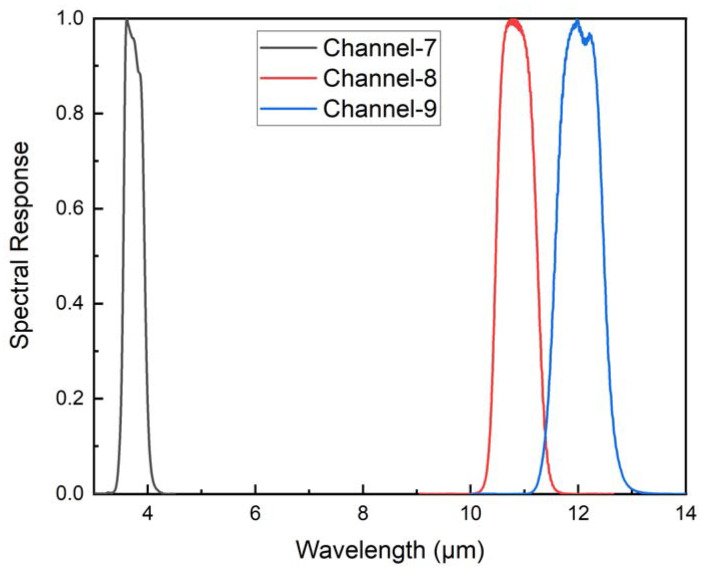
The spectral response functions of the MIR and TIR channels of the SLSTR data.

It should be noted that the emissivity, radiance, and atmospheric parameters are all related to the sensor channels and are obtained by integrating the value of a specific wavelength (*λ*) with the spectral response function (SLF). The calculation is shown in Equation (2), and the SLF of the MIR and TIR channels of the Sentinel-3 SLSTR sensor is shown in Figure 2 [28].

(2) $$P_{chn}=\frac{\int_{\lambda_{1}}^{\lambda_{2}}P\left(\lambda \right)f\left(\lambda \right)d\lambda }{\int_{\lambda_{1}}^{\lambda_{2}}f\left(\lambda \right)d\lambda }$$

where *P_chn_* is the channel parameters, *P*(λ) and *f*(λ) are the parameters and spectral response value at wavelength *λ*, and *λ*_1_ and *λ*_2_ are the upper and lower bounds of the wavelength of the channel, respectively.

### 2.2. Initial Values Estimation

One TIR SW algorithm and one MIR-TIR SW algorithm were developed based on the simulation dataset, TIR emissivity was retrieved using the NDVI-based method [24], and the LST was retrieved by the TIR SW algorithm. Then, the initial MIR emissivity can be obtained using the MIR-TIR SW algorithm after inputting the known TIR emissivity and LST.

#### 2.2.1. Development of SW Algorithms

The generalized SW algorithm [29] using two TIR channels (TIR SW), which linearizes the radiative transfer equation by assuming that atmospheric and land surface temperatures are not very different and that absorption is weak [30], corrects the LST errors using emissivity parameterization to achieve viewing angle-dependent LST retrieval [31]. The LST can be expressed in terms of a linear combination of brightness temperatures (BTs) of two TIR channels as in Equation (3).

(3) $$T_{s}=A_{0}+P\left(T_{i}+T_{j}\right)/2+M\left(T_{i}-T_{j}\right)/2$$

where *T_s_* is the LST, *T_i_* and *T_j_* are the BTs of TIR channels, *A*_0_ is a constant, and both *P* and *M* are functions of the emissivity, as shown in Equation (4).

(4) $$P=1+\alpha \frac{1-\bar{\varepsilon }}{\bar{\varepsilon }}+\beta \frac{\Delta \varepsilon }{{\bar{\varepsilon }}^{2}}\;M=\gamma^{\prime }+\alpha^{\prime }\frac{1-\bar{\varepsilon }}{\bar{\varepsilon }}+\beta^{\prime }\frac{\Delta \varepsilon }{{\bar{\varepsilon }}^{2}}$$

where *ε* and Δ*ε* is the averaged and differential values of TIR emissivity, *α*, *β*, *α*′, *β*′, and *γ*′ are coefficients. Moreover, a quadratic term for brightness temperature difference was added according to the LST error statistics [29], and then the TIR SW algorithm is shown in Equation (5).

(5) $$T_{s}=a_{0}+\left(a_{1}+a_{2}\frac{1-\bar{\varepsilon }}{\bar{\varepsilon }}+a_{3}\frac{\Delta \varepsilon }{{\bar{\varepsilon }}^{2}}\right)\left(\frac{T_{i}+T_{j}}{2}\right)+\left(a_{4}+a_{5}\frac{1-\bar{\varepsilon }}{\bar{\varepsilon }}+a_{6}\frac{\Delta \varepsilon }{{\bar{\varepsilon }}^{2}}\right)\left(\frac{T_{i}-T_{j}}{2}\right)+a_{7}{\left(T_{i}-T_{j}\right)}^{2}$$

where *a*_0_, *a*_1_,…, and *a*_7_ are the algorithm coefficients that can be fitted based on the simulation dataset. The algorithm has been utilized to produce MODIS LST products and has been successfully applied to other TIR remote sensing data [32,33], and the performance has been extensively validated and proven to achieve accurate LST results [34].

In addition, the three-channel split-window algorithm that included one MIR and two TIR channels [35] (MIR-TIR SW) was also developed. The MIR-TIR SW algorithm first proposed to retrieve LST from Geostationary Operational Environmental (GOES) satellite data, which was obtained by approximating the Planck function using a power function, linearizing the radiative transfer equation at the brightness temperature of the band and by regressing the fitted parameters that consider the atmospheric transmittance. After linearizing the radiative transfer equation around brightness temperature at surface level ($T_{i}^{*}$) and top-of-atmosphere ($T_{i}$) and approximating the Planck function with a power function [35], Equations (6) and (7) are obtained.

(6) $$T_{l}^{*}-T_{l}=\frac{1-\tau_{l}}{\tau_{l}}\left(T_{l}-T_{a}^{↑}\right)$$

(7) $$T_{s}-T_{l}^{*}=\frac{\left(1-\varepsilon_{l}\right)}{\varepsilon_{l}}\left[\frac{T_{l}^{*}}{n_{l}}+\frac{\left(n_{l}-1\right)}{n_{l}}\left(1-\tau_{l}\right)\cdot T_{l}^{*}-\left(1-\tau_{l}\right)\cdot T_{a}^{↓}\right]$$

where *T_s_* is the LST, *n_l_* is the fitted constants of the power function, *ε_l_* is the channel emissivity, *τ_l_* is the transmittance, and $T_{a}^{↑}$ and $T_{a}^{↓}$ are the mean radiative temperature of the atmosphere in the upward and downward direction, respectively. Combining the two equations gives Equation (8).

(8) $$T_{s}=C_{1l}T_{l}-C_{2l}T_{a}^{↑}-C_{3l}T_{a}^{↓}$$

(9) $$C_{*l}=k_{0}\left(*\right)+k_{1}\left(*\right)\frac{\left(1-\varepsilon_{l}\right)}{\varepsilon_{l}}$$

where *C*_1_*_l_*, *C*_2_*_l_*, and *C*_3_*_l_* are the functions consisting of *n_l_*, *ε_l_*, and *τ_l_*, and they can be expressed by Equation (9) using a regression method to find the appropriate coefficients *k*_0_ and *k*_1_ when the transmittance is not available. Then, the MIR-TIR algorithm form is shown in Equation (10).

(10) $$T_{s}=b_{0}+b_{1}T_{m}+b_{2}T_{i}+b_{3}T_{j}+b_{4}\frac{1-\varepsilon_{m}}{\varepsilon_{m}}T_{m}+b_{5}\frac{1-\varepsilon_{i}}{\varepsilon_{i}}T_{i}+b_{6}\frac{1-\varepsilon_{j}}{\varepsilon_{j}}T_{j}$$

where *T_m_*, *ε_m_* are the BT and emissivity of the MIR channel, *T_i_*, *ε_i_* and *T_j_*, *ε_j_* are that of the two TIR channels, and *b*_0_, *b*_1_,…, and *b*_6_ are the algorithm coefficients that can be also fitted using the simulation dataset.

#### 2.2.2. MIR Emissivity Estimation

The emissivity is required to be known to retrieve the LST using the SW algorithm; the TIR emissivity can be calculated as follows for the TIR SW algorithm [24]:

(11) $$\begin{array}{cc} NDVI<NDVI_{s} & \varepsilon =c+\sum e_{\lambda }\cdot \rho_{\lambda }\\ NDVI\geq NDVI_{v} & \varepsilon =\varepsilon_{v}+d\varepsilon \\ NDVI_{s}\leq NDVI\leq NDVI_{v} & \varepsilon =\varepsilon_{v}\cdot f+\varepsilon_{g}\cdot \left(1-f\right)+4\cdot d\varepsilon \cdot f\cdot \left(1-f\right) \end{array}$$

where *NDVI* is the normalized difference vegetation index which can be calculated by the reflectance of the red and near-infrared channels and *NDVI_s_* and *NDVI_v_* are the thresholds for non-vegetated (0.20) and fully vegetated (0.86) pixels. For the non-vegetated pixels, *ρ*_λ_ is the Sentinel-3 visible/near-infrared channel reflectances and *c* and *e*_λ_ are the empirical coefficients and the values are shown in Table 1. It can be seen that the fitted RMSEs for the TIR channels are 0.0174 and 0.0085, respectively, which is similar to the accuracy of the results of the previous study [1]. However, the RMSE of the fitting results for the MIR emissivity reaches 0.0572, which could be due to its high variability, and thus the NDVI method may not be suitable for the estimation of the MIR emissivity.

For the fully vegetated pixels, *ε_v_* is the emissivity of the vegetation component and *dε* is the emissivity increment caused by the cavity effect of multiple scattering [36]. For the mixed pixels, *ε_g_* is the emissivity of the background component, which can be obtained by TIR channel normalization [37] based on the land cover type-based look-up table from the previous study [24] as *ε_v_*, and *f* is the fraction vegetation coverage that can be estimated by the following equation:

(12) $$f={\left(\frac{NDVI-NDVI_{s}}{NDVI_{v}-NDVI_{s}}\right)}^{2}$$

Then, the LST can be retrieved by the TIR SW algorithm using the TIR emissivity. Based on the BTs of two TIR channels (*T_i_, T_j_*) and the MIR channel (*T_m_*), the known LST (*T_s_*), TIR emissivity (*ε_i_, ε_j_*), and algorithm coefficients (*b*_0_, *b*_1_,…, and *b*_6_) of the TIR-MIR SW algorithm, the initial value of the MIR emissivity (*ε_m_*) can be calculated as shown below after substituting into Equation (10).

(13) $$\varepsilon_{m}=\frac{b_{4}T_{m}}{\left[T_{s}-\left(b_{0}+b_{1}T_{m}+b_{2}T_{i}+b_{3}T_{j}-b_{4}T_{m}+b_{5}\frac{1-\varepsilon_{i}}{\varepsilon_{i}}T_{i}+b_{6}\frac{1-\varepsilon_{j}}{\varepsilon_{j}}T_{j}\right)\right]}$$

### 2.3. Emissivity Result Optimization

It is assumed that the atmospheric conditions are stable within a local spatial window of N*N pixels of the Sentinel-3 remote sensing image, implying that the three atmospheric parameters (downward radiance, upward radiance, and transmittance) are considered constant. For the parameters in the set of the N*N RTEs (Equation (14)) corresponding to N*N pixels within the local spatial window, the LSTs (*T_s_*) have been obtained by the TIR-SW algorithm, the initial values of MIR emissivities (*ε_m_*) have been estimated by Equation (13), and only the three atmospheric parameters (*L_m__↓_*, *L_m__↑_*, *τ_m_*) are unknown. When N*N is greater than the number of unknowns, then the least squares method was utilized, which can be used to find the best approximation of the atmospheric parameters by minimizing the error between the resulting calculated top-of-atmosphere radiance and the remote sensing observations.

(14) $$\left\{\begin{array}{c} L_{TOA}^{1}=\left[\varepsilon_{m}^{1}\cdot B\left(T_{s}^{1}\right)+\left(1-\varepsilon_{m}^{1}\right)\cdot L_{m↓}\right]\cdot \tau_{m}+L_{m↑}\\ L_{TOA}^{2}=\left[\varepsilon_{m}^{2}\cdot B\left(T_{s}^{2}\right)+\left(1-\varepsilon_{m}^{2}\right)\cdot L_{m↓}\right]\cdot \tau_{m}+L_{m↑}\\ \ldots \\ L_{TOA}^{N\times N}=\left[\varepsilon_{m}^{N\times N}\cdot B\left(T_{s}^{N\times N}\right)+\left(1-\varepsilon_{m}^{N\times N}\right)\cdot L_{m↓}\right]\cdot \tau_{m}+L_{m↑} \end{array}\right.$$

Then, the MIR emissivity of the pixels within the local spatial window was modified using the fitted atmospheric parameters as below, then through re-substituting into Equation (14) to update the atmospheric parameters. Iterating the process until the stop condition is satisfied, which is either that the change in the output of two consecutive iterations is small and the result is stable, or that the number of iterations reaches the upper limit, and the final MIR emissivity retrieval results were obtained.

(15) $$\varepsilon_{m}^{k}=\frac{\frac{L_{TOA}^{k}-L_{m↑}}{\tau_{m}}-L_{m↓}}{B\left(T_{s}^{k}\right)-L_{m↓}}$$

## 3. Experimental Results

The proposed method was used in the simulation dataset to evaluate the theoretical performance, and then applied to real Sentinel-3 SLSTR images to obtain the MIR emissivity retrieval results. Finally, the application accuracy is verified by cross-comparison with the MODIS MIR emissivity remote sensing product.

### 3.1. Retrieval of the Simulation Dataset

#### 3.1.1. LST Retrieval Results

To better simulate the real satellite observation situation, 0.05 K and 0.08 K noise equivalent differential temperatures (NEΔT) were added to the BTs of two TIR channels and one MIR channel based on the sensor evaluation result, separately [38]. Moreover, the uncertainties of 0.030, 0.015, and 0.015 were also included in the emissivity of each channel to present the possible estimation error according to the previous studies [1,37]. Then, the coefficients of the TIR and MIR-TIR SW algorithms were fitted based on the simulation dataset including noise for all parameters using multiple linear regression, and the LST RMSEs of two SW algorithms for different regions are shown in Table 2. It can be seen that both SW algorithms can retrieve the LST with RMSE < 1.50 K, while the MIR-TIR SW algorithm gives slightly more accurate LST results with RMSE differences of 0.04 K, 0.12 K, 0.25 K, and 0.07 K for different regions, respectively, and the errors become larger with the increase in CWV for both SW algorithms.

**Figure 3:**
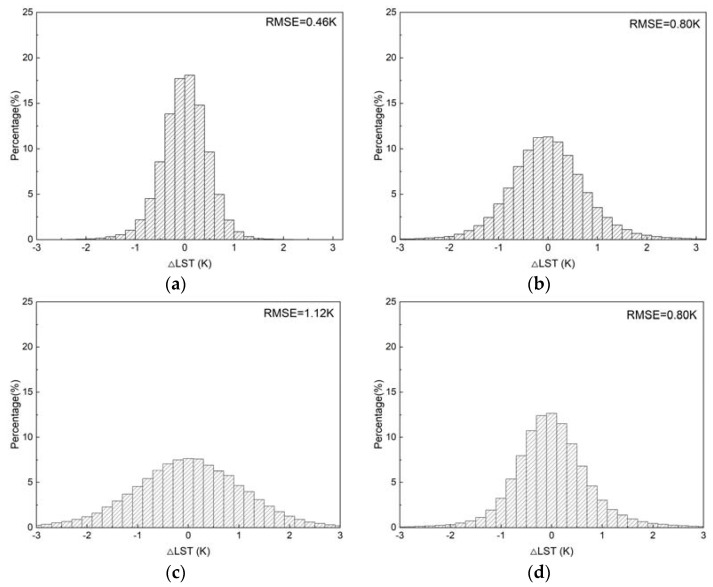
LST residuals histograms of the TIR SW algorithm. (**a**) Polar; (**b**) mid-latitude. (**c**) tropical; (**d**) overall.

The TIR SW algorithm was used to provide LST values for the calculation of MIR emissivity in Equation (13); the LST residual histograms for different regions are shown in Figure 3. It can be seen that the residual distribution approximates a normal distribution with mean values equal to 0, while approximately 98.60%, 90.94%, 82.43%, and 91.86% of the residuals fall within the [−1.0, 1.0] K interval, respectively. Overall, the TIR SW algorithm can retrieve the LST accurately and ensure the reliability of subsequent MIR emissivity calculations.

#### 3.1.2. MIR Emissivity Results

After obtaining the LSTs, the initial values of the MIR emissivity were calculated according to Equation (13), and then iterative optimization is applied to obtain the results using Equations (14) and (15). The RMSEs of MIR emissivity for different regions are shown in Table 3, the errors in the initial values calculated directly are relatively large, with an RMSE of 0.055 for global atmospheric conditions and all local RMSEs above 0.045. This could be due to the LST retrieval using the TIR channel being more than twice as sensitive to errors in emissivity than the MIR channel [9], which makes the MIR emissivity more affected by the LST errors.

**Figure 4:**
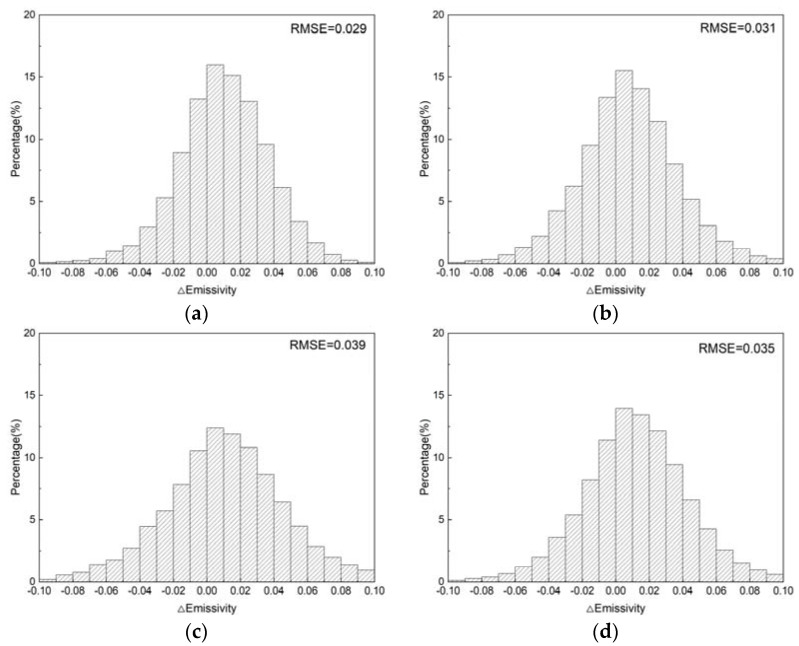
MIR emissivity residuals histograms of the proposed method. (**a**) Polar; (**b**) mid-latitude; (**c**) tropical; (**d**) overall.

After optimization, all the RMSEs were reduced to within 0.04, with a maximum of 0.039 (tropical), and a minimum of 0.029 (polar), which is comparable to the accuracy validated in the previous studies [39,40] and outperforms the results of applying the NDVI method for the TIR channel to the MIR channel in Section 2. The residual histograms are shown in Figure 4, the distribution is close to a normal distribution with a mean value of 0, while 78.06%, 70.13%, 59.18%, and 64.52% of the residuals are within the interval [−0.03, 0.03] under different regions of the atmosphere, respectively. This demonstrates the effectiveness of the iterative optimization method and the ability to achieve better results under dry atmospheric conditions.

### 3.2. Sentinel-3 SLSTR Images Application

**Figure 5:**
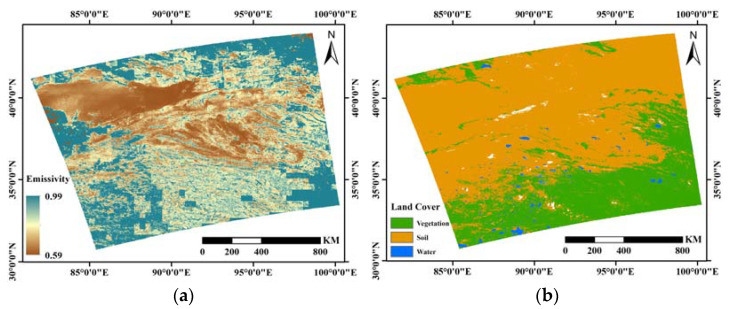
Application results of the study area. (**a**) The MIR emissivity retrieval image; and (**b**) the land cover map.

The proposed method was applied to one Sentinel-3 SLSTR image that was observed on 5 July 2022, 15:37:49 UTC, and the study area is in Western China and includes a variety of land cover types with soil, vegetation, and water. The MIR emissivity retrieval image is shown in Figure 5a and exhibits significant land surface heterogeneity with data ranging from 0.59 to 0.99, and the land cover map (Figure 5b) is also obtained based on the MODIS land cover type product (MCD12Q1) to present the variation between different classes. For the different locations, the emissivity in the central part of the study area is lower than in other regions, while for the different land cover types, vegetation and water have higher emissivity than soil. The average emissivities, found in the statistical results based on the land cover types, are 0.927 for soil, 0.968 for vegetation, and 0.971 for water, respectively, which is consistent with the theoretical values of the samples in the simulation dataset.

**Figure 6:**
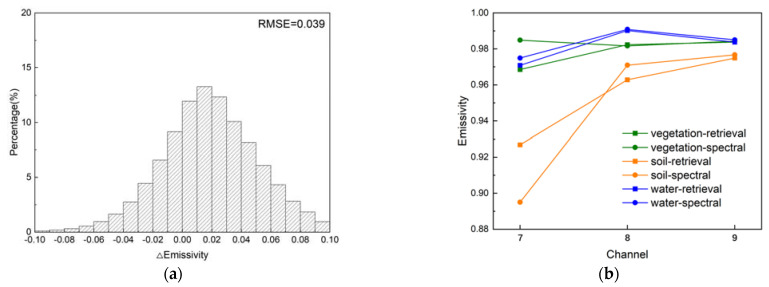
The validation results of the retrieval image. (**a**) The emissivity residual histogram; and (**b**) comparison of emissivity spectral shape.

The MODIS MIR emissivity product (MOD11B1) was utilized to validate the accuracy of the retrieval image, an evaluation result of RMSE = 0.039 was achieved and the residual histogram is shown in Figure 6a. The histogram shows a normal distribution with a total of 57.74% of the residuals within the interval [−0.03, 0.03], which is generally consistent with the validation results based on the simulation dataset, and comparable accuracy was achieved without relying on multiple source remote sensing images and external data. In addition to the numerical comparison, the shape of the emissivity band spectra was also considered. The average emissivity curves of the three land cover classes in the retrieval results were compared with the spectral curves in the simulation dataset, and the results are shown in Figure 6b. It can be seen that the vegetation has the greatest variation, probably because different properties such as morphology and water content may produce variations in emissivity, followed by soil, and the water has the most stable shape and the closest retrieval result. In general, the emissivity shapes of the three land cover classes in the retrieval results vary with the channel in patterns that are generally consistent with the spectral library data. Furthermore, the variability of the spectra of different land cover types in the MIR channel was also taken into account. The statistical significance test for the difference in MIR emissivity between different classes was carried out, and the results showed that the emissivities between the pixels of the three land cover types shown in Figure 5b are significantly different from each other. The results of the cross-validation and significance test prove the effectiveness of the proposed method, which is beneficial for further analysis of LST retrieval or land surface spectrum using the MIR channel.

## 4. Discussions

### 4.1. Relationship between the Errors of LSTs and Emissivity

According to the principle of the proposed method, the accuracy is directly influenced by the results of the SW algorithms. According to the previous study [9], the relationship between the errors of LSTs and emissivity can be derived from the Planck function as Equation (16):

(16) $$\frac{\partial T_{s}}{\partial \varepsilon_{\lambda }}=\frac{T_{s}}{\varepsilon_{\lambda }}\cdot \frac{\exp\left(c_{2}/\lambda T_{s}\right)-1}{c_{2}/\lambda T_{s}\cdot \exp\left(c_{2}/\lambda T_{s}\right)}$$

where *T_s_* is the LST, *ε_λ_* is the spectral emissivity, *λ* is the wavelength, and *c*_2_ is the radiance constant of the Planck function.

**Figure 7:**
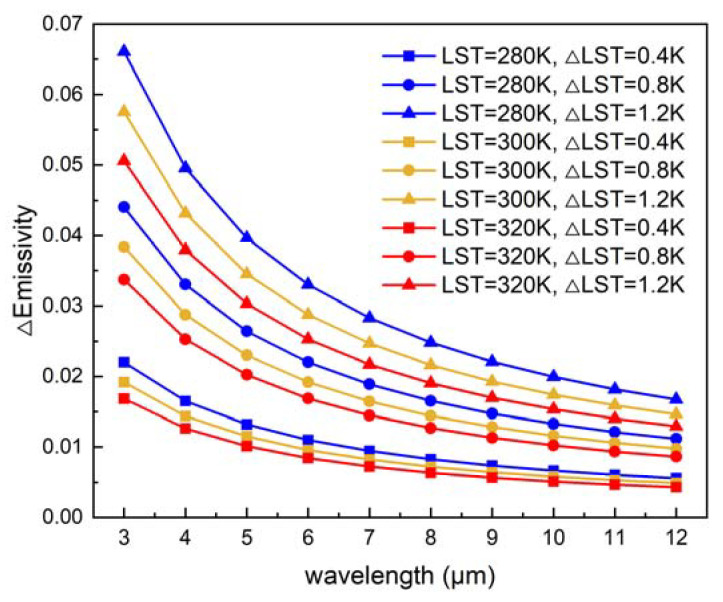
Errors of emissivity as a function of wavelength.

The quantitative relationship between the errors of emissivity and wavelength is shown in Figure 7; it can be seen that the errors decrease with increasing wavelength and increase with increasing LST errors. When the LST is 300 K, the LST noise of 0.4, 0.8, and 1.2 K, may lead to emissivity errors of 0.014, 0.029, and 0.043 at 4 µm, respectively. In addition, at 3 μm, the emissivity errors increase to 0.019, 0.038, and 0.058. Moreover, with the same LST error, the errors of the emissivity in the cold cases are larger. For example, the emissivity errors caused by LST noise of 0.4, 0.8, and 1.2 K increase to 0.017, 0.033, and 0.050 at 4 µm, when the LST is 280 K. Therefore, the RMSE in the polar region in Table 3 is larger than in the other regions. As can be seen from Figure 6, most errors in the retrieval result of MIR emissivity are within 0.03, which would cause approximately 0.8 K uncertainty at the LST of 300 K.

### 4.2. Future Improvements of the Proposed Method

The influence of the view zenith angle (VZA) is not fully taken into account by the proposed method. The local zenith angle of the oblique view of the SLSTR data is close to 55°, which may cause angular effects resulting in emissivity variations [41], as well as atmospheric parameters [33] due to differences in transmission paths. This could cause the atmospheric and land surface parameters in the simulation dataset to not represent the actual observations well, reducing the accuracy of the LST and the emissivity results. Therefore, fitting the SW algorithm coefficients separately for different VZAs, and increasing the angular normalization of the observed radiance at the top of the atmosphere will help to improve performance.

The CWV is another key factor to be considered in the proposed method. The error of the SW algorithm increases with the CWV [32], which will directly affect the estimation accuracy of the initial value of MIR emissivity. Therefore, the experiments were conducted in relatively dry atmospheric conditions in this paper. The use of more robust LST estimation methods, such as the hybrid algorithm that requires no additional input [42,43,44], will be applied to enable the algorithms to be suitable to a wider range of atmospheric conditions. Moreover, the empirical relationship between solar radiance and CWV has been summarized in previous studies [45]. So, the applicability of the proposed method can be extended to all-day remote sensing images by estimating CWV using the TIR channel correlation-based algorithm, such as the modified split-window covariance-variance ratio (MSWCVR) algorithm [46], and thus eliminating solar radiance in MIR channels observed during the daytime without introducing additional data.

As the iterative optimization is performed in an image window with constant atmospheric conditions, there may be patches of spatial discontinuity in the retrieval image of MIR emissivity, and the selection of a suitable filtering method to enhance continuity is considered. Finally, cross-comparison with the MODIS MIR emissivity product, the spectral shape similarity and differential significance in emissivity between different land cover types were used for accuracy validation, and more ground-measured data will be introduced to further illustrate the effectiveness of the proposed method.

## 5. Conclusions

In this paper, a practical MIR emissivity retrieval method was proposed based on the principle of radiance transfer from the nighttime Sentinel-3 SLSTR remote sensing image. The TIR channel-based SW algorithm and the TIR and MIR channel-based SW algorithm were combined to provide the initial values of the MIR emissivity, and the spatial information provided by the assumption based on local region atmospheric stability was used for iterative optimization by the RTE. The proposed method was investigated using the simulation dataset built on multiple land surface samples and global atmospheric profiles, and experimental results under different atmospheric conditions show that the new method has accurate theoretical performance. The method was also applied to the Sentinel SLSTR image of China observed on 5 July 2022, and by comparing the land cover types in the study area, the MIR emissivity image has a reasonable spatial distribution and the numerical relationships between various land types are consistent with the simulation dataset. Moreover, cross-comparison results with the MODIS MIR emissivity product showed that the RMSE of the retrieved image is 0.039, which is useful for follow-up studies in several applications.

## Figures and Tables

**Table 1:** Empirical coefficients for estimating the emissivity.

Channel	*c*	*e*_1_	*e*_2_	*e*_3_	*e*_4_	*e*_5_	*e*_6_	RMSE
7	1.0043	−0.2230	−1.0291	1.3553	−0.2443	0.2590	−0.9229	0.0572
8	0.9368	0.0721	−0.2294	0.1593	−0.0179	0.0646	0.0009	0.0174
9	0.9638	0.0674	−0.2542	0.2387	−0.0861	0.0369	0.0051	0.0085

**Table 2:** LST RMSEs of two SW algorithms for different regions.

SW Algorithm	Polar	Mid-Latitude	Tropical	Overall
TIR	0.46	0.80	1.12	0.80
MIR-TIR	0.44	0.70	0.89	0.75

**Table 3:** RMSEs of MIR emissivity for different regions.

MIR Emissivity	Polar	Mid-Latitude	Tropical	Overall
Initial values	0.053	0.045	0.048	0.055
Optimized results	0.029	0.031	0.039	0.035

## Data Availability

SLSTR images supporting the reported results can be found on and downloaded from the website (https://scihub.copernicus.eu/) accessed on 7 November 2022, and the MODIS images supporting the reported results can be found on and downloaded from the website (https://www.earthdata.nasa.gov/) accessed on 7 November 2022.
